# Ageing is associated with a decline in peripheral blood CD56^bright ^NK cells

**DOI:** 10.1186/1742-4933-3-10

**Published:** 2006-11-29

**Authors:** Shivani M Chidrawar, Naeem Khan, Y L Tracey Chan, Laxman Nayak, Paul AH Moss

**Affiliations:** 1 Medical School, University of Birmingham, Edgbaston, Birmingham, B15 2TT, UK; 2 Institute of Cancer Studies, Vincent Drive, University of Birmingham, Edgbaston, Birmingham, B15 2TT, UK; 3 Centre for Applied Gerontology, Selly Oak Hospital, Raddlebarn Road, Birmingham, B29 6JD, UK

## Abstract

**Background:**

Natural killer (NK) cells are cytotoxic lymphocytes that lack CD3 and express variable levels of CD16, CD56 and CD57. In recent years NK cells have been categorised into two major groups based on the level of CD56 expression. This phenotypic classification correlates with functional activity as CD56^bright ^NK cells are the major cytokine producing subset whereas CD56^dim ^NK cells exhibit greater cytotoxic activity. Previous studies have revealed a reduction in total NK cell numbers in association with ageing and this study sought to determine the potential influence of ageing on the number of NK cell subsets within peripheral blood.

**Results:**

The number of NK (CD56^+^CD3^-^) cells within peripheral blood did not change with increasing age. The number of CD56^dim ^NK cells also remained stable with ageing. In contrast the absolute number of CD56^bright ^NK cells within peripheral blood declined by 48% with ageing from a mean of 15.6/μl in individuals aged 20–40 years to 8.1/μl in those aged 60+ years (*p = 0.0004*).

**Conclusion:**

The number of CD56^bright ^NK cells within peripheral blood declines with age. As this population plays a central role in cytokine secretion during the innate immune response this decline may contribute to impaired immune regulation in elderly individuals

## Background

Natural killer (NK) cells are a heterogenous group of large granular lymphocytes derived from bone marrow precursors which are phenotypically distinct from T and B lymphocytes. NK cells have been recognised to constitute up to 10% of peripheral blood lymphocytes (PBMC) and are also found in peripheral tissues including the peritoneal cavity, placenta and liver [1,2]. NK cell activation is governed by a balance of signals through activatory and inhibitory receptors [3] although activation in response to downregulation of MHC class I molecules on the target cell is a potent influence. NK cells represent the first line of defence against virus-infected and neoplastic cells and initiate cell lysis and cytokine production in the absence of prior antigenic stimulation [1,2,4]. The potential role of NK cells in the control of cancer has been suggested by the observation of an inverse correlation between the NK cell count and incidence of malignant disease in cohort studies of humans with further support derived from murine studies [5-7]. NK cell participation has been reported in the immune response to several viruses including HIV, hepatitis B and C, but it may have particular relevance in host defence against herpes viridae [7-9]. The relevance of NK cells in the host defence against herpes viruses is best illustrated in the case of isolated NK cell deficiency resulting in a range of systemic herpes viral infections [10].

The NK cell phenotype is characterized by lack of expression of the CD3 complex together with variable expression of CD16, CD56 and CD57. CD56 is an integrin with homotypic adhesion properties and in recent years studies have demonstrated the existence of two NK cell subsets based on the intensity of CD56 expression. These CD56^bright ^and CD56^dim ^subsets are thought to differ in their tissue homing properties due to differential patterns of expression of chemokine receptors and adhesion molecules. CD56^bright ^NK cells lack or express low levels of CD16 and represent only 10% of circulating NK cells but are the dominant NK cell subset within lymph nodes [11]. In contrast, the major CD56^dim ^NK cell population has a granular phenotype and exhibits more cytotoxic activity than CD56^bright ^NK cells. In addition this subset expresses higher levels of CD16 and is thus more potent in antibody-directed cell cytotoxicity (ADCC). In contrast, CD56^bright ^NK cells are the primary source of immunoregulatory cytokines such as IL-10, IL-13 and GM-CSF following antigen recognition with minimal production of these molecules being observed from CD56^dim ^NK cells [3]. This cytokine response is likely to be critical in the activation of the adaptive immune response by NK cells.

Ageing is associated with an increased mortality and morbidity from infectious disease and cancer and this is at least partly related to the development of immune senescence. Previous reports have demonstrated alterations in NK cell number and phenotype in association with age. In this study we have extended these observations to enumerate NK cells with respect to the CD56^bright ^and CD56^dim ^subsets, and have correlated these findings with age.

## Results

### Patient characteristics

**Figure 1:**
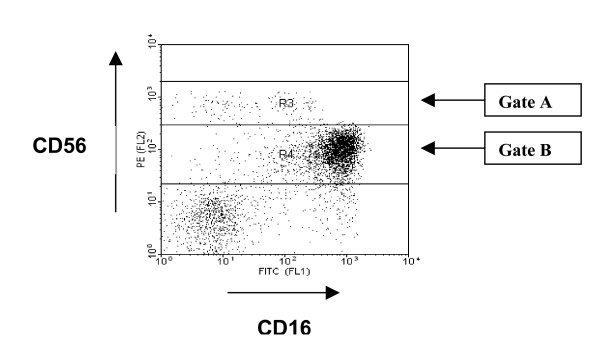
**Identification of CD56^bright ^and CD56^dim ^NK cells**. Peripheral blood lymphocytes were stained with CD3, CD56 and CD16 mABs and analysed. The CD3^negative ^lymphocyte population was initially gated and then analysed for CD56 and CD16 expression. Gates A and B allow enumeration of the CD56^bright ^and CD56^dim ^NK cell population respectively.

The study population consisted of 115 individuals that were divided into those aged 20–40 years (n = 25), 40–60 years (n = 23) and > 60 years (n = 67, mean age 79 years). The absolute lymphocyte count was determined using an automated Coulter full blood counter and expressed as cells/μl of blood. The total NK population was expressed both as an absolute number (μl/blood) and as a percentage of the lymphoid pool. CD56 expression on NK cells was determined by staining cells with an anti-CD56 antibody conjugated to phycoerythrin followed by flow cytometry. NK cells were separated into two subsets based on the level of CD56 expression and cell numbers determined in a similar way (figure 1).

### The effect of age on NK cell number: elderly individuals have a trend towards a greater proportion of NK cells within peripheral blood

**Figure 2:**
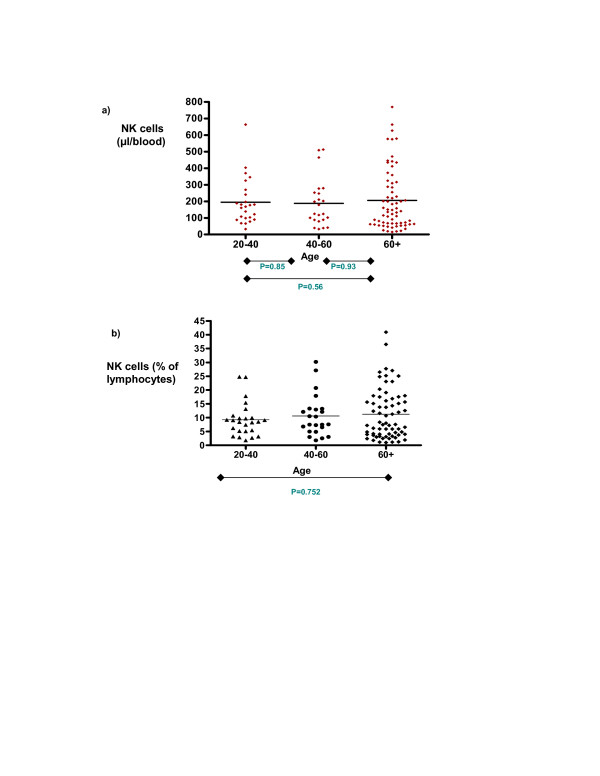
**The effect of age on peripheral blood NK cells**. These graphs illustrate (a) the number of NK cells μl/blood and (b) the percentage of lymphocytes that NK cells represent within samples in each age group; 20–40(n = 25) 40–60(n = 23) and 60 + (n = 67). The mean values within each respective cohort group are marked. Statistical analysis was carried out as described in the Methods; p values between groups are displayed and results are summarised further in table 1.

The number of NK cells was determined for each age cohort and compared as a function of ageing. A marked variation in the number of NK cells was observed between different donors. However, although there was a trend towards a small increase in mean NK cell number with ageing this did not reach statistical significance (195 cells/μl for individuals aged 20–40 years; 206 cells/μl for donors aged > 60 years) (figure 2). The total lymphocyte count fell with age and analysis of NK cells as a percentage of lymphocytes revealed a small increase with advancing age (table 1). NK cells represent 9.3% of the peripheral lymphocyte pool in donors aged 20–40 years which increases to 11.2% in donors aged over 60 years.

**Table 1:** The effect of age on NK cell number

**Age(yrs)**	**N =**	**Mean NK cell μl/blood (+/- SD)**	**Mean NK cells % of lymphocytes (+/-SD)**
**20–40**	25	194.9 (+/- 140.4)	9.25 (+/- 6.1)
**40–60**	23	187.6 (+/- 144.5)	10.59 (+/- 7.5)
**> 60**	67	205.8 (+/- 184.0)	11.22 (+/- 9.1)

Statistical analysis was carried out as described in the Methods; no significant differences between groups were found.

### The effect of age on CD56^bright ^NK cell number: elderly individuals have a significantly lower number of CD56^bright ^NK cells

**Figure 3:**
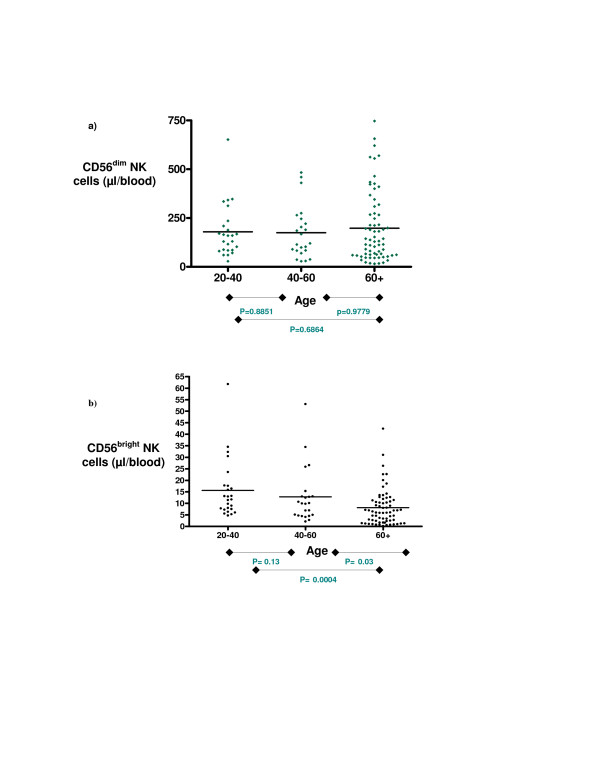
**The effect of age on peripheral blood CD56^bright ^and CD56^dim ^NK cell number**. These graphs illustrate (a) the number of CD56^dim ^NK cells μl/blood and (b) the percentage of CD56^bright ^NK cells μl/blood within samples in each age group; 20–40(n = 25) 40–60(n = 23) and 60 + (n = 67). The mean values within each respective cohort group are displayed. Statistical analysis was carried out as described in the Methods and p values between groups are shown. These results are summarised together with the percentage of the lymphoid pool occupied by CD56^bright ^and CD56^dim ^cells and are displayed in table 2.

The effect of age was analysed with respect to the number of peripheral blood CD56^bright ^NK cells using 3 colour flow cytometric analysis. A progressive decrease is observed with advancing age; 15.6 cells/μl detected in the younger cohort aged 20–40 years falling to 8.1 cells/μl in donors aged over 60 years (p = 0.0004) (figure 3) (table 2). CD56^bright ^NK cells analysed as a percentage of the peripheral blood lymphocytes also significantly decreases with advancing age.

**Table 2:** The effect of age on CD56^bright ^and CD56^dim ^NK cells

**Age**	**20–40**	**40–60**	**60+**
**N =**	25	23	67
**CD56**^**bright **^**μl/blood (+/- SD)**	15.64 (+/-12.8)	12.81 (+/-12) ***	8.13 (+/-7.9)****
**CD56**^**bright **^**as % of lymphocytes (+/-SD)**	0.76 (+/-0.6)	0.72 (+/-0.6) *	0.50 (+/-0.5) **
**CD56**^**dim **^**μl/blood (+/-SD)**	179.3 (+/-135.3)	174.8 (+/-135)	197.7(+/-180.3)
**CD56**^**dim **^**as % of lymphocytes (+/-SD)**	8.49 (+/-5.8)	9.86 (+/-7.0)	11.7 (+/-9.3)

Statistical analysis was carried out as described in the Methods; * p = 0.031 between the 40–60 and 60+ age groups, ** p = 0.012 between the 20–40 and 60+ age groups. ***p = 0.03 between the 40–60 and 60+ age groups, ****p = 0.0004 between the 20–40 and 60+ age groups.

### The effect of age on CD56^dim ^NK cell number: cell number remains constant with age

**Figure 4:**
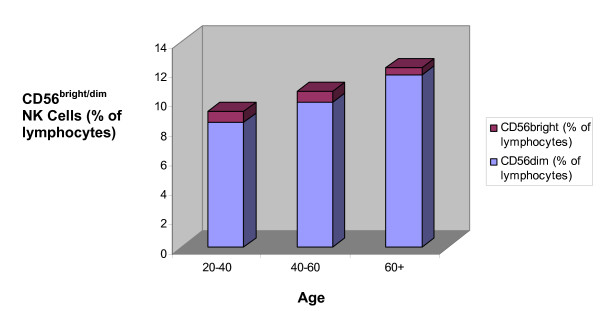
**The percentage of total peripheral blood lymphocytes represented by CD56^bright ^and CD56^dim ^NK cells with advancing age**. This graph illustrates the percentage of the total peripheral blood lymphoid pool represented by CD56^bright ^and CD56^dim ^NK cell subsets. These results are also documented in tables 1 and 2.

The influence of age on peripheral blood CD56^dim ^NK cells revealed relatively consistent numbers at all ages. A trend towards a slight increase in CD56^dim ^NK cells μl/blood was found between the 40–60 and 60+ year cohorts although this did not reach statistical significance (figure 3). A non-significant increase in CD56^dim ^NK cells as a percentage of the peripheral blood lymphoid pool was also observed with advancing age (table 2 , figure 4).

## Discussion

The decline in immune function with increasing age is termed immune senescence and leads to impaired responses to vaccination, an increased incidence of autoimmune disorders and increased morbidity and mortality to infectious disease [12-15]. Immune senescence has been attributed to a number of factors including thymic involution and memory T cell accumulation resulting in contraction of the T cell repertoire. More recently, debate has focused on the role of innate cellular immunity with regard to impaired immune function. Remarque *et al*. have reported that elderly individuals with low NK numbers have a three-fold increased risk of mortality in the first two years of follow up compared to those with high NK cells [16]. Functional studies have measured NK cell activity against the K562 tumour cell line and demonstrate impaired NK cell cytotoxicity in elderly donors [17]. Further evidence is derived from studies on centenarians, regarded as a model example of healthy ageing, who have been reported to have well preserved NK cell cytotoxicity [18]. Our data shows that NK cell numbers are relatively stable with advancing age but as the total peripheral blood lymphocyte count decreases there is a small increase in the proportion of the lymphoid compartment occupied by NK cells.

Our findings demonstrate that the number of CD56^bright ^NK cells declines with advancing age which may have considerable implications for NK cell function in the elderly cohort. This decline was apparent across all three age groups indicating a gradual decline with healthy ageing. The CD56^dim ^NK cell subset remains relatively constant but occupies a greater portion of the peripheral blood lymphoid pool with advancing age. Our findings are in keeping with Krishnaraj (1997) who also reports a significant reduction in the proportion of CD56^bright ^cells with relative sparing of the CD56^dim ^subset [19]. This contrasts with Borrego *et al*. (1999) who reports an expansion in the proportion in CD56^dim ^cells but little change in CD56^bright ^NK cells [20]. Differences in the methodology employed in these studies are likely to account for the variation in results.

NK cell associated cytotoxic function is thought to be mediated primarily through the CD56^dim ^NK cell subset whereas the CD56^bright ^NK cells can be thought of primarily as a cytokine producing subset. The CD56^dim ^cell is more cytotoxic than the CD56^bright ^NK cell subset [21] and the morphological appearance of CD56^dim ^cells shows greater granularity [1]. A substantial body of data indicates that the CD56^bright ^subpopulation plays a critical role in the early innate immune response. CD56^bright ^NK cells have a higher proliferative capacity than CD56^dim ^NK cells [22] and are also the primary source of NK cell-derived cytokines including TNF-α, IFN-γ, IL-10, IL-13, and GM-CSF [11]. NK cell culture with lipopolysaccharide-activated macrophages, providing an endogenous monokine source, resulted in a greater than 6-fold IFN-y production from CD56^bright ^NK cells in comparison with CD56^dim ^NK cells [11].

The importance of NK cells in the priming of adaptive immunity is becoming clearer in recent years and CD56^bright ^NK cells play an important role in the activation of dendritic cells [23]. A recent study found that lipopolysaccharide cultured with immature dendritic cells induced proliferation of peripheral blood NK cells restricted to CD56^bright ^NK cell subset which was also the main source of IFN-γ associated with dendritic cell interaction [24]. CD56^bright ^NK cells also interact with monocytes in a reciprocal fashion thereby promoting inflammation [25].

CD56 expression also determines the anatomical location of the major NK cell subsets. CD56^bright ^NK cells are the predominant NK cell subset within lymphoid tissue and inflammatory lesions and exhibit differential expression of a range of chemokine receptors and adhesion molecules including CD62L, CCR7 and CXCR3 [24]. The CD56^bright ^NK cell subset constitutively expresses high affinity IL-2R and produces IFN-γ in response to picomolar doses of IL-2 from T cells through interactions within secondary lymphoid organs [11].

These observations suggest that a reduction in CD56^bright ^NK cells may contribute to the impairment in immunity towards newly encountered foreign pathogens that is associated with ageing. The role of NK cells in the initiation of adaptive T and B cell responses [26] may be particularly impaired in elderly individuals due to lower circulating numbers of the CD56^bright ^NK cells.

## Conclusion

We have shown that the number of CD56^bright ^NK cells within peripheral blood decreases with age. As this population plays a critical role in the cytokine response of the innate immune system these findings suggest that a decline in CD56^bright ^NK cell number may contribute to the development of immune senescence.

## Methods

### Subjects

67 donors aged over 60 were contacted through the 'Thousand Elders', Centre of Applied Gerontology, Selly Oak Hospital. Peripheral blood was acquired through venepuncture into the following vacutainer tubes (BD Vacutainer); Sodium Heparin, EDTA, and Clot activator (silica coated, BD Haemoguard). Informed consent was obtained from all donors under the South Birmingham ethical committee protocol. Peripheral blood from a further 48 individuals were acquired from the National Blood Service, Blood Transfusion Services, Birmingham. These samples were anonymous, such that only the sex and age range between 5–10 years was provided. These samples were kept in EDTA tubes (BD Vacutainer) and collected on the same date of peripheral blood collection. All blood samples were submitted to the Queen Elizabeth Hospital Haematology lab for full blood count analysis on the same date of collection (in EDTA tubes).

### Isolation of peripheral blood mononuclear cells

Isolation of peripheral blood mononuclear cells (PBMC's) from heparinized blood samples was achieved through Ficoll-assisted (Lymphoprep, Axis Shield UK) density gradient centrifugation. Cell washing was carried out twice using RMPI media (RPMI 1640, GibcoBRL). Cells were subsequently resuspended in 1 ml freezing media (10% DMSO, 90% heat activated FCS). Aliquots of cells were frozen down in 1 ml cryotubes at concentrations of around 1–10 × 10^6 ^cells/ml. Cryotubes were placed in a Nalgene Cryo Freezing container and stored at immediately at -80°C. Isolation and freezing of PMBC's was undertaken on the same day of blood collection. Isolation of PBMC's used for functional work however were resuspended in RPMI growth media.

### Staining with monoclonal antibodies and FACS analysis

Frozen cells were thawed in waterbaths no longer than 1 min, and subsequently washed twice in MACS buffer. Cells were incubated at 4°C for 25 minutes with CD3-PC5 (Cyto-Stat Coulter Clone), CD56-PE (Dako Cytomation), CD16-FITC (Dako Cytomation). Cell populations were analysed using Win MDI 2.8. following data collection using a Coulter XL flow cytometer.

### Statistical analysis

Statistics were calculated using Graph Pad Prism4 software. The Mann-Whitney U Test was employed to find inter-group differences. Two-tailed p values were employed, where a significant result was represented by p = 0.05 or less.

## Competing interests

The author(s) declare that they have no competing interests.

## Authors' contributions

SMC was involved in the design, acquisition, analysis and interpretation of data and also drafted the manuscript. NK was involved in the design of the study, interpretation of data and revision of the manuscript. YLTC contributed to the design of the study and interpretation of experiments. LN was involved in the recruitment of elderly donors to the study. PAHM conceived the study, and was involved in the interpretation of data and revisions to the manuscript. All authors have read and approved the final manuscript.
